# Supplementary material for: Low irradiance multiphoton imaging with alloyed lanthanide nanocrystals
Source: Nat Commun. 2018 Aug 6;9:3082. doi: 10.1038/s41467-018-05577-8 (PMC6079005; doi:10.1038/s41467-018-05577-8)
Supplement: Supplementary file 1 — Supplementary Information [file 41467_2018_5577_MOESM1_ESM.pdf]

Supplementary Information

Low irradiance multiphoton imaging with alloyed lanthanide nanocrystals

B. Tian, et al.

TABLE OF CONTENTS

Supplementary Figure 1. Nanocrystal sizing and monodispersity
Supplementary Figure 2. Nanocrystal characterization by TEM
Supplementary Figure 3. Nanocrystal morphology by X-ray diffraction
Supplementary Figure 4. Upconverted emission spectra from single UCNPs
Supplementary Figure 5. $NaEr_{0.2}Yb_{0.8}F_4$ and $NaEr_{0.6}Yb_{0.4}F_4$ core/shell power-dependent emission
Supplementary Figure 6. Minimum excitation intensities required to image single UCNPs
Supplementary Figure 7. Low irradiance imaging of single aUCNP nanocrystals and ensembles
Supplementary Figure 8. Single UCNP absorption cross sections and quantum yields
Supplementary Figure 9. Upconverting lifetime measurements
Supplementary Figure 10. Optical set-up and aberration correction
Supplementary Figure 11. Selection of single nanocrystals
Supplementary Table 1. Precursor injection volumes for 4-nm shell growth
Supplementary Methods: Lifetime measurements
Supplementary Methods: Kinetic simulations
Supplementary Table 2. General simulation parameters
Supplementary Table 3. Judd-Ofelt parameters and reduced matrix elements
Supplementary Figure 12. Modeling output of steady-state Er^{3+} manifold populations

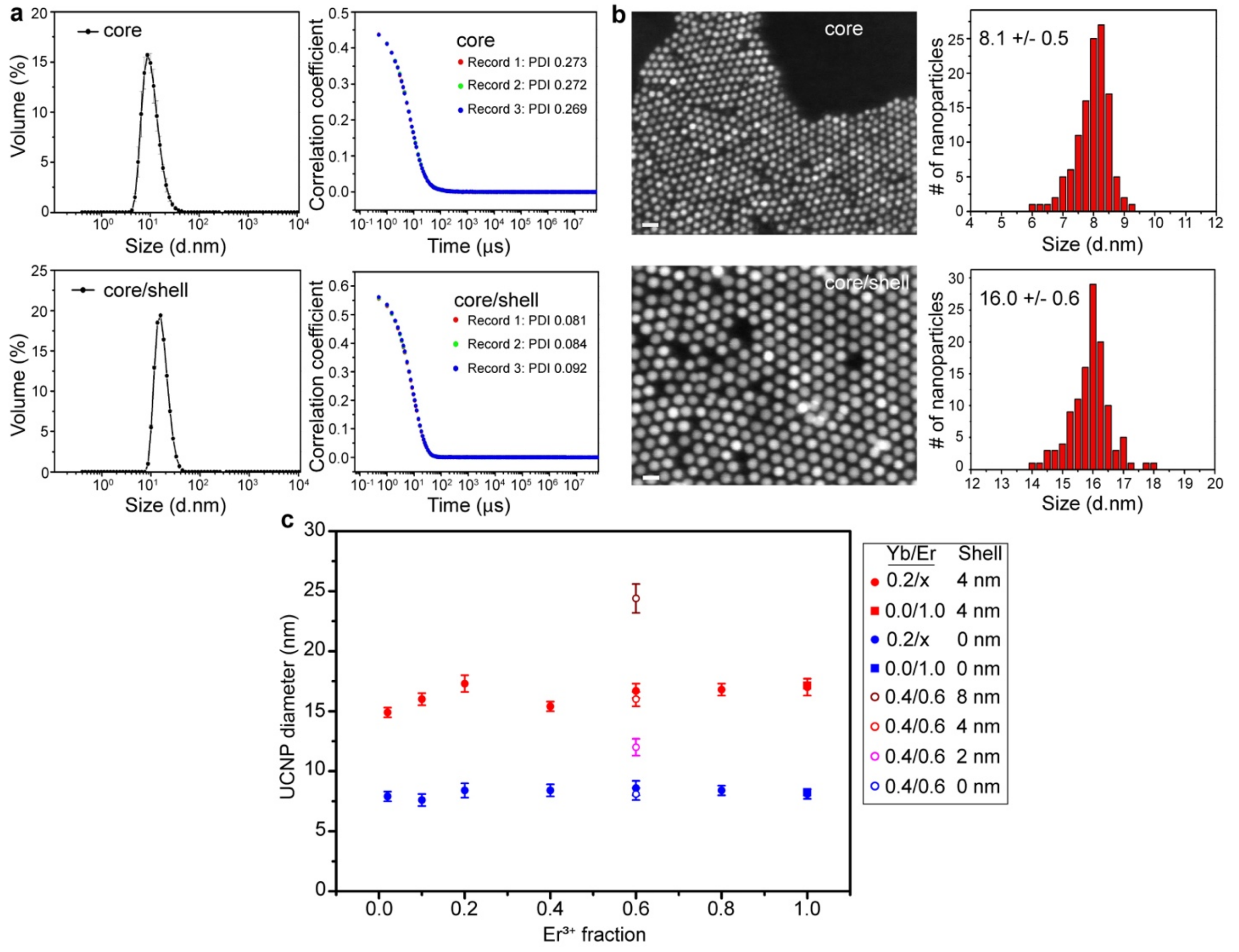

Supplementary Figure 1. Nanocrystal sizing and monodispersity. a UCNPs size distributions characterized by DLS. Size distributions are $NaEr_{0.6}Yb_{0.4}F_4$ core and core/shell aUCNPs, respectively, with correlation curves of 3 parallel measurements. Data were collected at 25 °C with nanoparticles dispersed in hexane. PDI, polydispersity index. **b** UCNPs size distributions characterized by STEM, of $NaEr_{0.6}Yb_{0.4}F_4$ core and core/shell aUCNPs. Statistics are mean ± one standard deviation ($n \geq 100$). **c** Monodispersity of nanoparticles synthesized in this study. UCNP cores (blue) synthesized to be 8 nm and overgrown $NaYF_4$: 20% Gd^{3+} epitaxial shells (red) to be 4 nm, or other size as in the legend. Statistics are mean ± one standard deviation ($n \geq 100$).

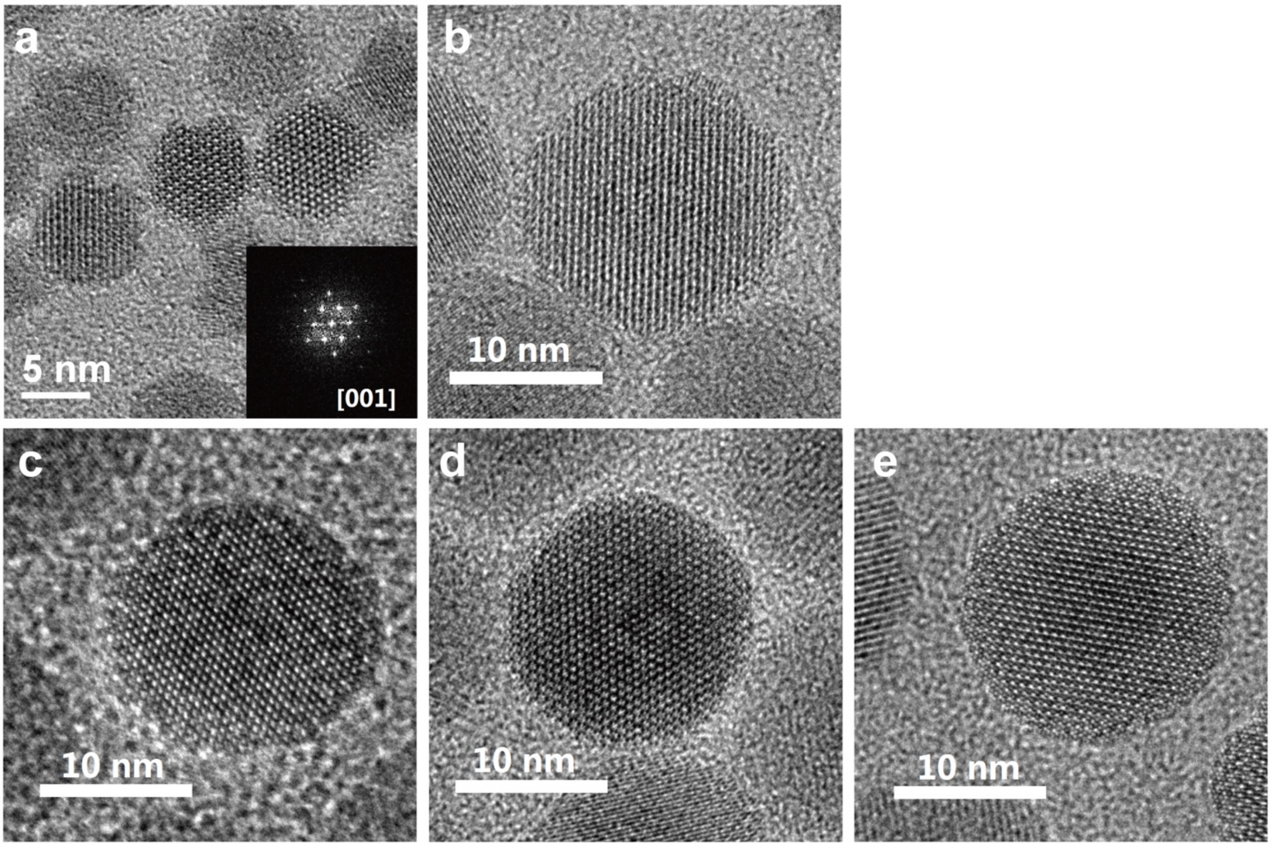

Supplementary Figure 2. Nanocrystal characterization by TEM. a High-resolution transmission electron microscope (HR-TEM) image of $NaEr_{0.6}Yb_{0.4}F_4$ aUCNPs. Inset: Fast Fourier transform (FFT) of the crystal structure from the center nanoparticle. **b-e** HR-TEM images of core/shell nanoparticles with different Ln^{3+} components: **b** $NaYF_4$: 20% Yb^{3+}, 20% Er^{3+}, **c** $NaEr_{0.2}Yb_{0.8}F_4$, **d** $NaEr_{0.6}Yb_{0.4}F_4$, and **e** $NaErF_4$. Images were taken at 200k X magnification, acceleration voltage 200 kV.

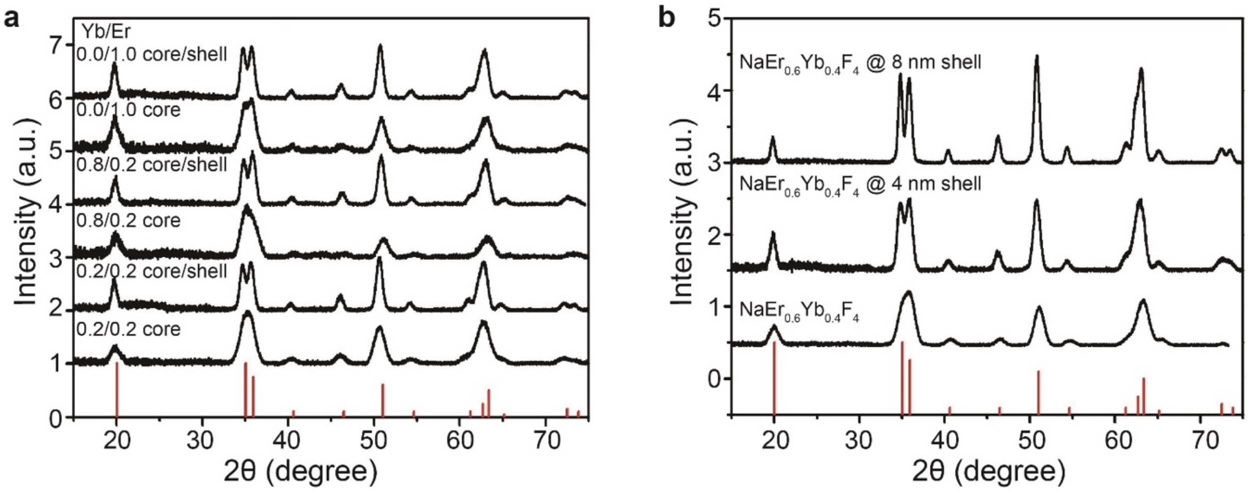

Supplementary Figure 3. Nanocrystal morphology by X-ray diffraction. **a** XRD patterns of UCNPs with varying Ln^{3+} content. **b** XRD patterns of $NaEr_{0.6}Yb_{0.4}F_4$ with different shell thicknesses. Red lines are Rietveld fitting for β phase $NaErF_4$ (JCPDS 27-0689). XRD patterns were measured with Co Kα radiation (λ= 1.78897 Å).

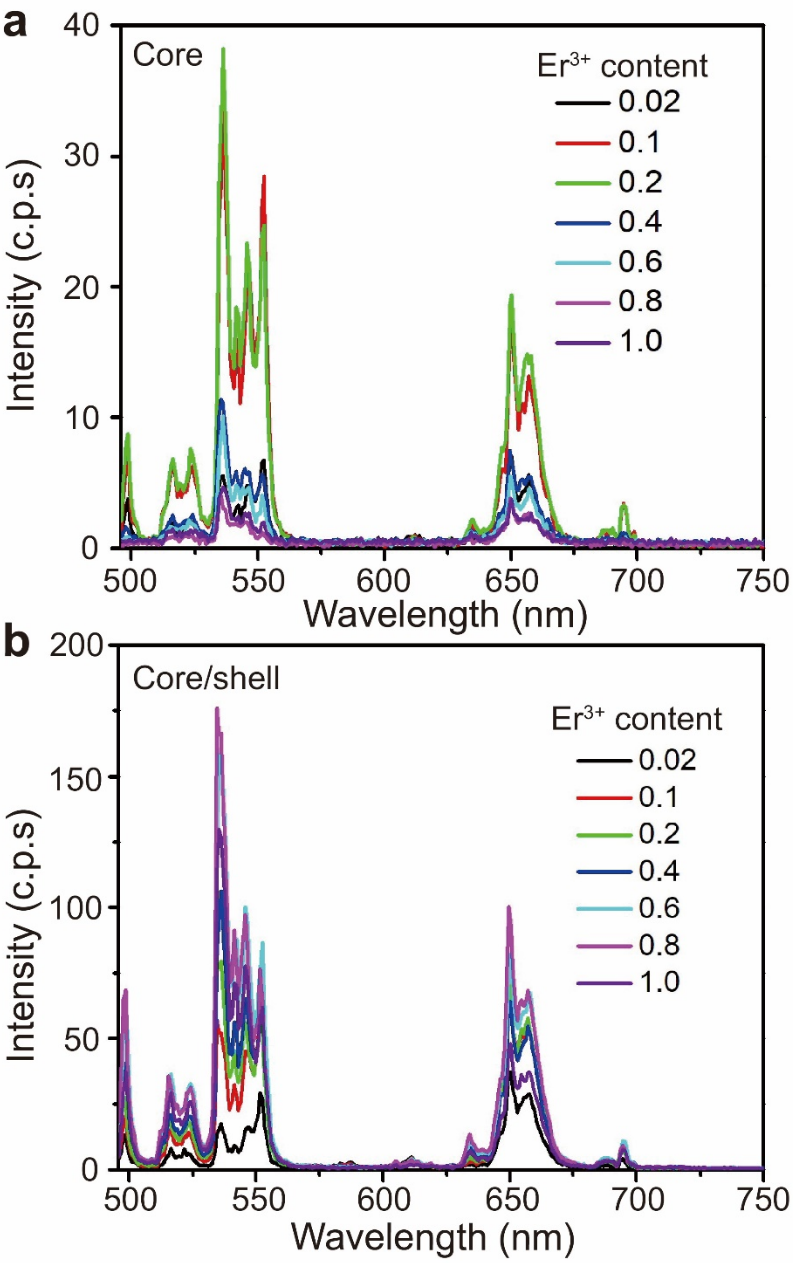

Supplementary Figure 4. Upconverted emission spectra from single UCNPs. Emission spectra from single (**a)** core and (**b)** core/shell UCNPs. Excitation density is 7.5 x 10^5 W cm^{-2}.

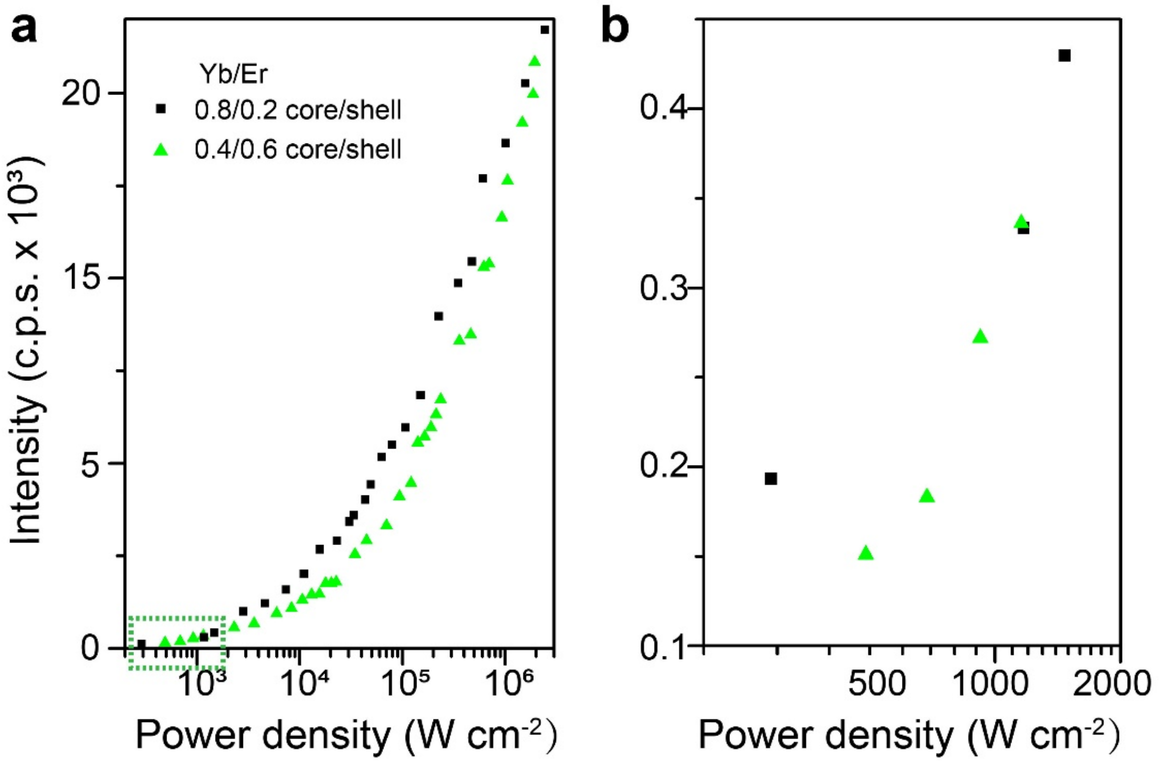

Supplementary Figure 5. Optimized core/shell aUCNP emission as a function of laser power density. **a** Visible emission (496 – 745 nm) of single core/shell $NaEr_{0.2}Yb_{0.8}F_4$ and $NaEr_{0.6}Yb_{0.4}F_4$ aUCNPs as a function of 980 nm laser excitation density. **b** Expansion of emission at low irradiance from highlighted area (green dashed frame).

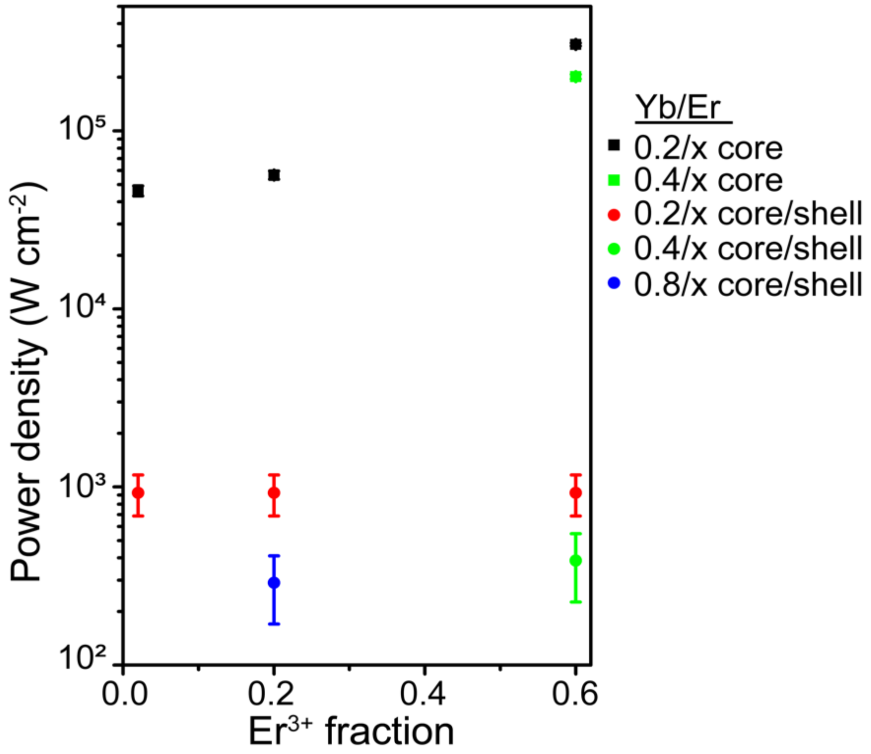

Supplementary Figure 6. Minimum excitation intensities required for imaging single UCNPs. The minimum excitation power densities to image 100 c.p.s. above background for UCNP cores and core/shells, corresponding to a signal:noise > 3. Error bars are standard deviation (*n* > 10).

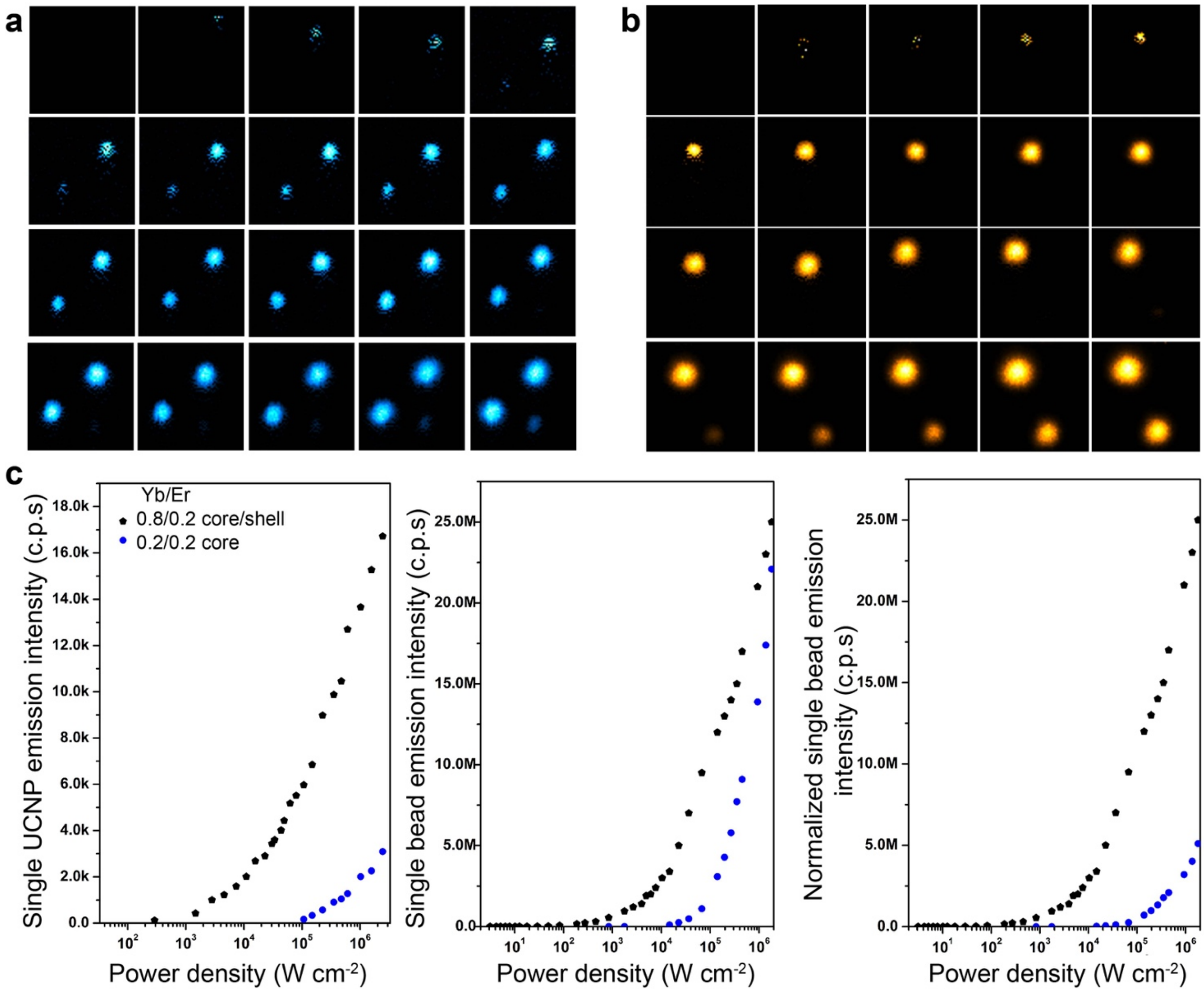

Supplementary Figure 7. Low irradiance imaging of single aUCNP nanocrystals and ensembles. a Imaging of aUCNPs as single nanocrystals. The minimum excitation power densities to image signal:background ≥ 3 for $NaEr_{0.2}Yb_{0.8}F_4$ core/shell aUCNPs (brighter upper right and bottom left spots) and $NaYF_4$: 20% Yb^{3+}, 20% Er^{3+} UCNPs (dimmer bottom right spot) are 290 and 1.07 x 10^5 W cm^{-2}, respectively. Power densities are (serpentine from top left) from 290 to 2.4 x 10^6 W cm^{-2}. **b** Imaging of aUCNP ensembles in polystyrene beads. The minimum excitation power densities to image and $NaEr_{0.2}Yb_{0.8}F_4$ aUCNP and $NaYF_4$: 20% Yb^{3+}, 20% Er^{3+} UCNP beads are 4.2 and 840 W cm^{-2}, respectively. Power densities are (serpentine from top left) 3.1 to 1.8 x 10^6 W cm^{-2}. Image color scales were generated by ImageJ adjusted to maximum intensity. **c** Single and ensemble UCNP emission intensity as a function of power density. Power dependent emission of (left) single aUCNPs, (middle) single beads, and (right) single beads normalized to 1,500 UCNPs per bead for both $NaYF_4$: 20% Yb^{3+}, 20% Er^{3+} core and $NaEr_{0.2}Yb_{0.8}F_4$ core/shell ensembles. Intensities were obtained from linecuts in **a** and **b**.

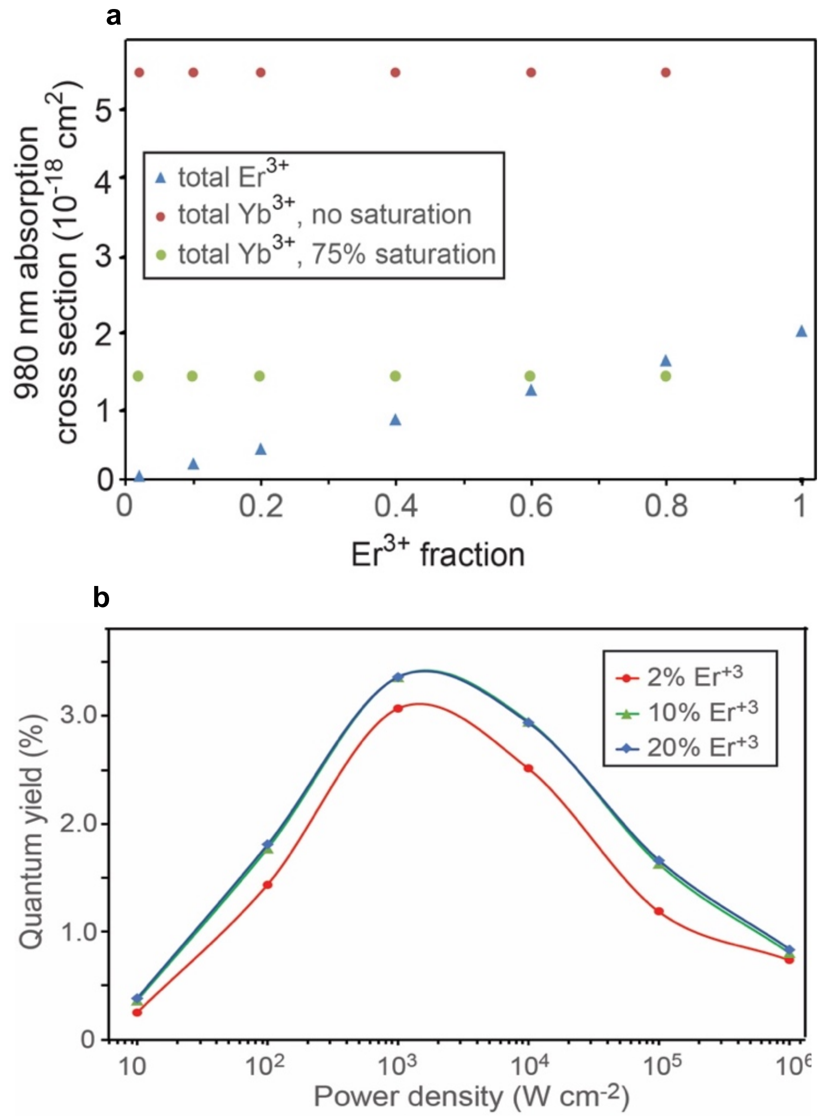

Supplementary Figure 8. Single UCNP absorption cross sections and quantum yields. a Calculated Er^{3+} and Yb^{3+} contributions to 8-nm UCNP absorption cross sections (σ_{980}) as a function of Er^{3+} content. Yb^{3+} fraction is 0.2 except for $NaErF_4$. Values were calculated as in Methods. **b** QY variation of core/shell UCNPs with 20% Yb^{3+} as a function of laser power density. Values were calculated as in Methods.

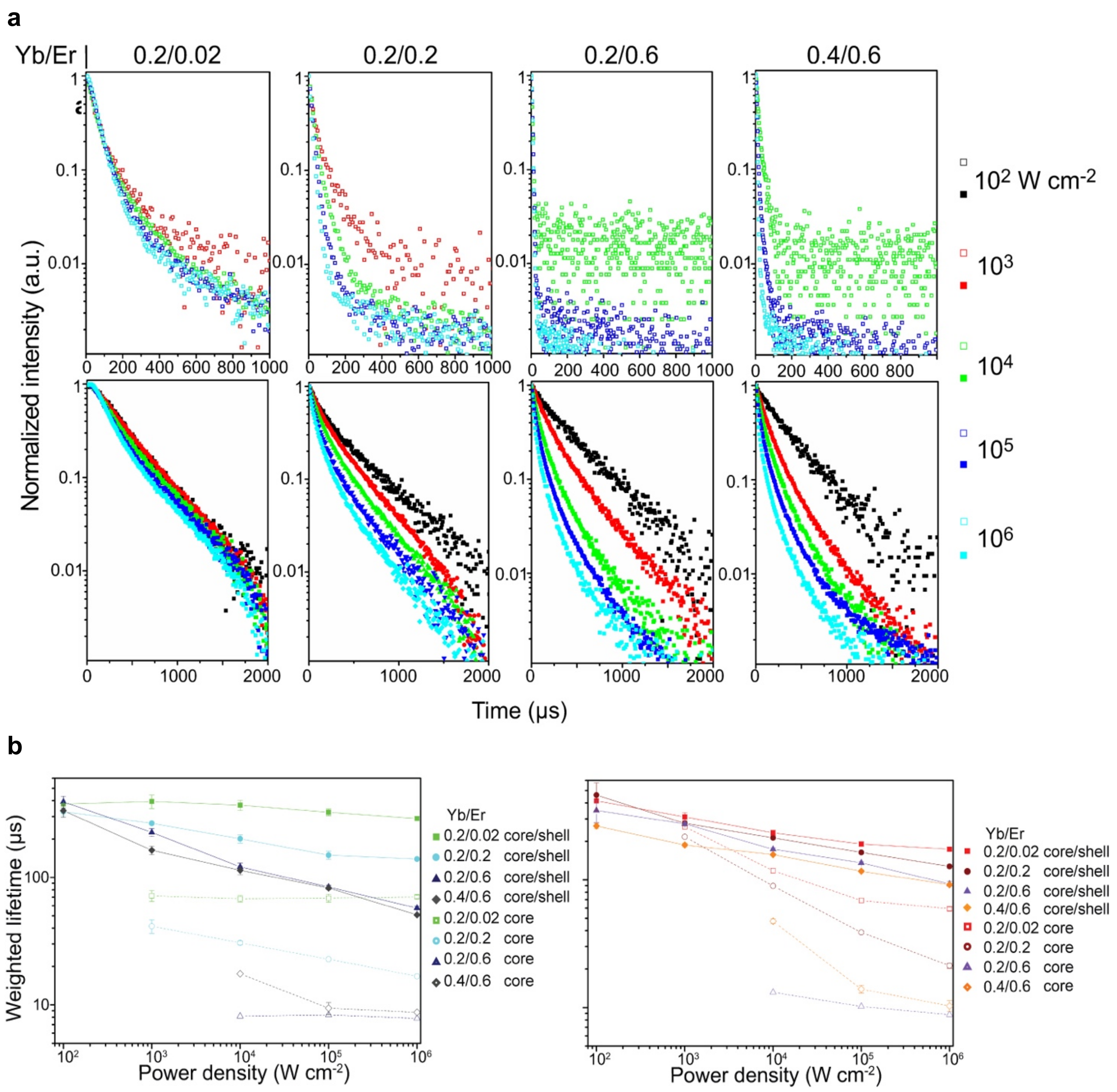

Supplementary Figure 9. Power dependence of upconverted lifetimes. a Lifetime decay curves of UCNPs at different excitation densities, of 8-nm cores and core/shell UCNPs with 4-nm shells. **b** UCNP weighted lifetimes at different power densities. Weighted lifetime of green ($^2H_{11/2}$, $^4S_{3/2}$) and red ($^4F_{9/2}$) Er^{3+} emission, obtained from exponential fitting of decay curves from **a**. Error bars are standard deviation propagated from uncertainties in exponential fittings and in some cases are smaller than data points.

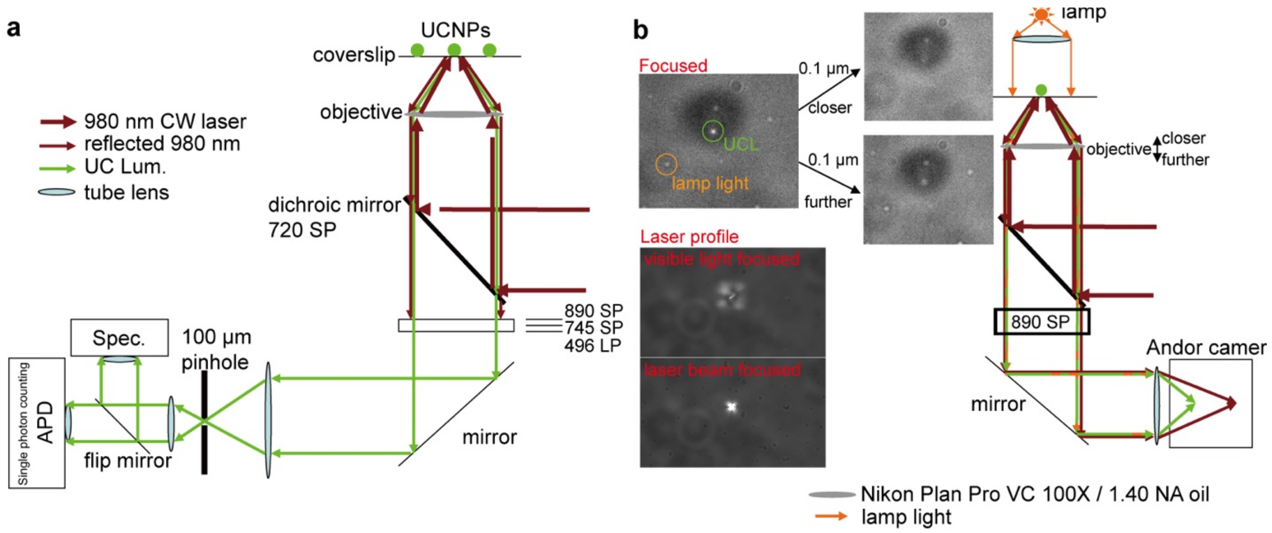

Supplementary Figure 10. Optical set-up and and aberration correction. a Experimental setup for single UCNP optical characterization. 980 nm continuous-wave (CW) laser from the back aperture of a 1.40 NA 100x Objective (Nikon), focused on the UCNPs through a No.1 glass coverslip. Reflected laser light is blocked by a combination of a 720-nm dichroic mirror and an 890-nm SP filter (Semrock). Upconverted emission passes through the objective, filters (745-nm shortpass and 496-nm longpass, Semrock) and a 100-μm pinhole, and then directed to either a single photon counting APD (MPD) or a liquid N_2 cooled CCD spectrometer (Princeton). For the lifetime setup, band pass filters (535/30 for green or 665/45 with 532 longpass for red) are used along with a time-correlated single photon counter (TCSPC, PicoHarp) which tags the photon arrival times of the collected luminescence with respect to the laser operating in pulsed mode modulated by a function generator. **b** Chromatic aberration check for objective and optics. To measure 980 nm chromatic aberration through a Nikon 100X/1.40 NA oil objective, visible light is directed through the same path and captured by an EMCCD (Andor). Upper set of images show UCNP emission and lamp light are focused at the same focal plane (to ± 0.1 μm), indicating that the focal planes of 980 nm laser and UCNP visible emission are the same. Objective is corrected for chromatic aberration. Upon removal of the laser filter after focusing the UCNP emission, the shape of the laser indicates it is not focused at this point. Image showing the shape of the focused 980 nm laser.

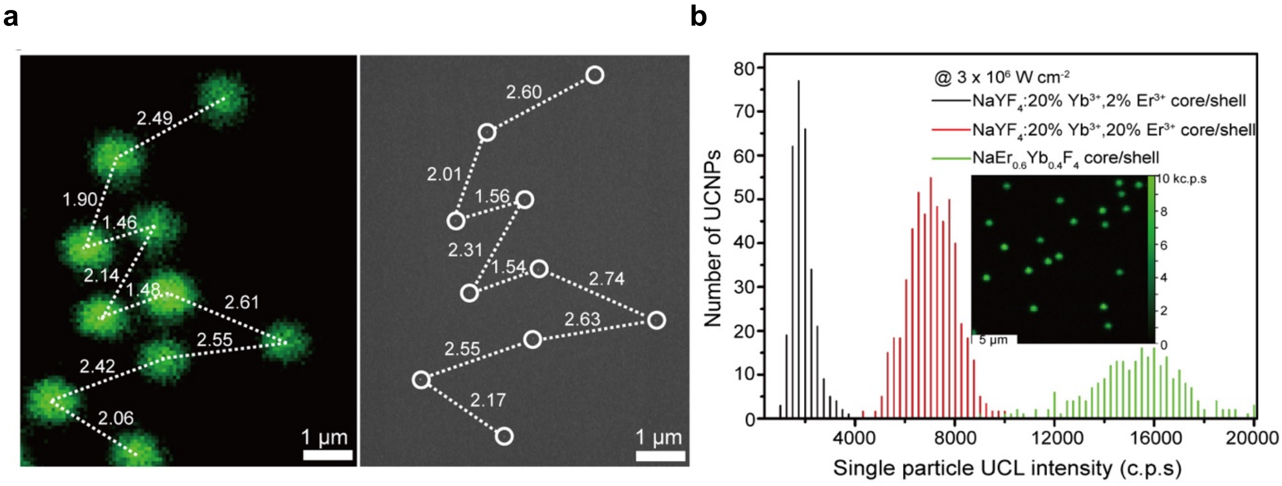

Supplementary Figure 11. Selection of single nanocrystals. a Correlated optical and STEM imaging of single UCNPs. Single 8-nm $NaYF_4$: 20% Yb^{3+}, 20% Er^{3+}, 20% Gd^{3+} UCNPs with 4 nm shell are dropcasted on a silicon nitride grid. Confocal image (left) is acquired before transferring to STEM imaging (right). **b** Spot intensities for single UCNP measurements. Emission histogram of $NaYF_4$: 20% Yb^{3+}, 2% Er^{3+}, 20% Gd^{3+} core/shell UCNPs, $NaYF_4$: 20% Yb^{3+}, 20% Er^{3+}, 20% Gd^{3+} core/shell UCNPs, and $NaEr_{0.6}Yb_{0.4}F_4$ core/shell aUCNPs. Data are obtained by counting visible emission intensities from ~300 upconverted luminescent spots in confocal images as shown in the insert confocal image of $NaYF_4$: 20% Yb^{3+}, 20% Er^{3+} core/shell UCNPs. Scanning area is 20 x 20 μm^2, resolution/ pixel size is 156 x 156 nm^2, and integration time is 25 ms for each pixel.

Supplementary Table 1. Precursor injection volumes for 4-nm shell growth

	1	2	3	4	5	6	7	8
Y/Gd-OA (mL)	0.28	0.36	0.44	0.52	0.62	0.72	0.83	0.95
Na-TFA-OA (mL)	0.14	0.18	0.22	0.26	0.31	0.36	0.42	0.48

Supplementary Methods: Lifetime measurements

To obtain lifetime data, a time-correlated-single-photon-counter (TCSPC, PicoHarp) was used to tag photon arrival times of collected luminescence with respect to the laser operating in pulsed mode. The resulting time-resolved luminescence plots were fit to exponential decays, and a weighted lifetime was calculated by $\tau_{\mathrm{eff}} = \frac{\sum \mathrm{A_i} * \tau_\mathrm{i}}{\sum \mathrm{A_i}}$ where τ_{eff} is the weighted lifetime, A_i are weighting factors, τ_i are radiative lifetimes, obtained from fitting of the decay curve.

Mean first exit times were calculated using $\tau_{exit}(r) = \frac{R^2 - r^2}{6D}$, where R is the radius of the nanoparticle, and r is the initial radial position of the excited state. D is the diffusion coefficient, which is calculated as $D = \frac{1}{2}\left(\frac{4\pi}{3} n_a\right)^{4/3} C_{dd}$, where $\mathrm{n_a}$ is the population of the acceptor (ground state) species, and C_{dd} is the energy transfer microparameter. The mean exit time can be integrated over all r in a nanoparticle to give $\langle \tau_{exit} \rangle_{NP} = \frac{R^2}{15D}$. This value corresponds to the average time for the excitation energy for a random ion excited in a nanoparticle to reach the surface of the nanoparticle for the first time. For simplicity, reported exit times assume that the acceptor population was equal to the ion concentration, which is valid at low fluences. At higher fluences, n_a will decrease, resulting in longer mean exit times.

Supplementary Methods: Modeling

Based on a previous model, we set up and numerically integrate systems of differential rate equations that model the change in population of each manifold (excited state) for the Yb^{3+} and Er^{3+} ions. This model does not assume a given mechanism, but instead computationally accounts for all transitions in the solution.

Supplementary Table 2. General simulation parameters

Parameter	***Value***
Simulation time period (ms)	3
Phonon energy (cm^{-1})	450
W^0_{MPR}, zero-phonon relaxation rate(s^{-1})[1]	$2·10^7$
α, MPR rate constant(cm)[2]	$3.5·10^{-3}$
Index of refraction (β-$NaYF_4$)	1.5
Volume per potential dopant site (nm^3)	$7.2395·10^{-2}$
Minimum dopant distance, β-$NaYF_4$ (nm)	0.3867
Absorption fwhm (cm^{-1})	400
Incident excitation wavelength (nm)	978

Kinetic simulations were performed according to the method of Chan *et al*. [ref JPCB 2012] using Igor Pro 6.3 (Wavemetrics). *N* ordinary differential equations, which represent the population of each of the *N* manifolds in the simulated system, were solved numerically using the Igor Pro Backwards Differentiation Formula integration method. All ions (i.e., Er^{3+} and Yb^{3+}) were placed in their ground states at the start of the simulation. Time steps for iterations were determined dynamically by the integration algorithm, and all simulated systems reached steady state by the end of the simulation time period. Lifetimes were simulated by performing a second simulation in which the excitation power density was set to zero, and initial manifold populations were set to the steady state populations of the previous simulation.

These simulations calculate and utilize the rates of all possible transitions, even those far from resonance. Since the radiative electric dipole transitions are calculated using Judd-Ofelt theory, all absorption transitions, even excited state absorption, are considered. In other words, for all initial and final states, *i* and *f*, the model incorporates the transition rates for all combinations of *i* and *f*, for all species. Likewise, all energy transfer (ET) processes are considered by the model – all ET processes with rates above a given threshold are incorporated into the differential equations to be solved. Therefore, back transfer is incorporated as simply another energy transfer process.

Supplementary Table 3. Judd-Ofelt parameters and reduced matrix elements

**Parameter**	**Er^{3+}**	**Yb^{3+}**
Ω_2 (10^{-20} cm^2)	2.11	N/A
Ω_4	1.37	

Ω_6	1.22	
S_{ED} Electric dipole Line strength (cm^2)		$3\cdot10^{-20}$
Source, Ω_λ	experimental	
Source, $\|\langle i\|U^\lambda\|j\rangle\|^2$	Kaminski, *Crystalline Lasers* (1996).	

Yb^{3+}: Since Yb^{3+} only has one excited state manifold, Judd-Ofelt parameters cannot be determined empirically from absorption spectra. However, the absorption cross section of Yb^{3+} in various fluoride matrices at the incident excitation wavelength (978 nm) has been reported by several sources to be in the range of $1\cdot10^{-20}$ cm^2 (ref: Deloach & Krupke, IEEE JOURNAL OF QUANTUM ELECTRONICS, VOL. 29. NO. 4, APRIL 1993), which agrees with the common observation that the absorption cross section of Yb^{3+} is an order of magnitude greater than that of the Er^{3+} ${}^4I_{15/2}\rightarrow{}^4I_{11/2}$ transition. With a peak width (fwhm) of ~400 cm^{-1}, the integrated cross section of the Yb^{3+} ${}^2F_{7/2}\rightarrow{}^2F_{5/2}$ transition is ~$5\cdot10^{-18}$ cm, resulting in an electric dipole line strength, S_{ED}, of approximately $3\cdot10^{-20}$ cm^2.

Er^{3+}: For simulations, the 34 lowest Er^{3+} manifolds (up to 51,200 cm^{-1}) were used.

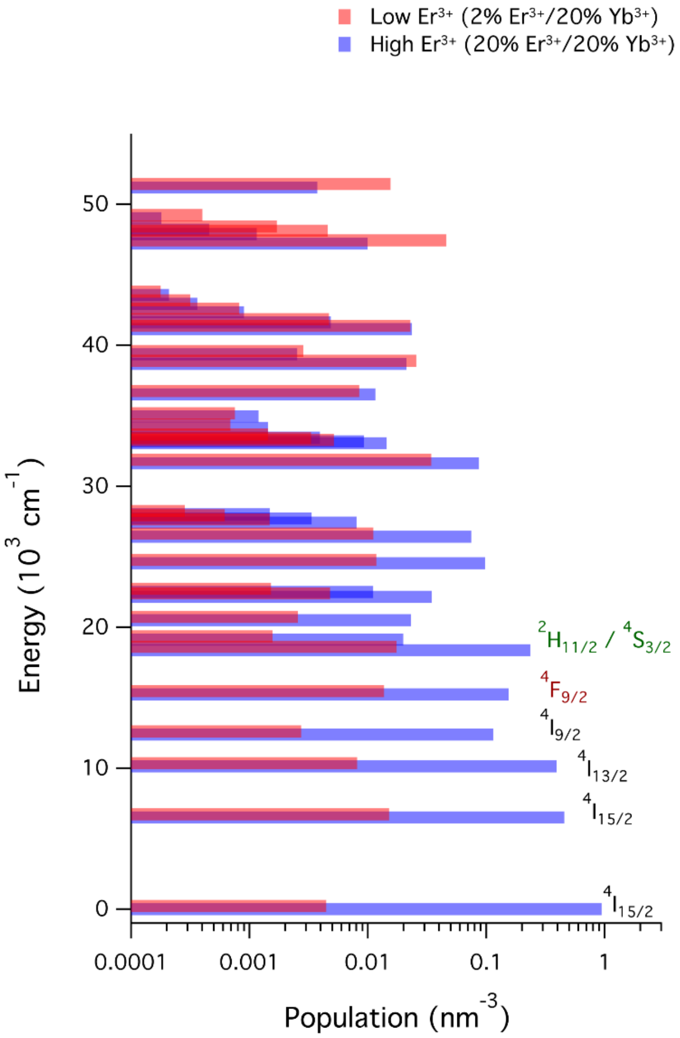

Supplementary Figure 12. Modeling output of steady-state Er^{3+} manifold populations. Populations are from differential rate equation calculations of 8-nm UCNPs with 20% Er^{3+} (blue bars) and 2% Er^{3+} (red bars), each with 20% Yb^{3+}, at 10^6 W cm^{-2} excitation.
